# Tumor Necrosis Factor Receptors: Pleiotropic Signaling Complexes and Their Differential Effects

**DOI:** 10.3389/fimmu.2020.585880

**Published:** 2020-11-25

**Authors:** Portia Gough, Ian A. Myles

**Affiliations:** Epithelial Therapeutics Unit, National Institute of Allergy and Infectious Disease, National Institutes of Health, Bethesda, MD, United States

**Keywords:** epithelial to mesenchymal transition, NF-kappa B, signaling/signaling pathways, TNF, TNF receptor, TNF blockade

## Abstract

Since its discovery in 1975, TNFα has been a subject of intense study as it plays significant roles in both immunity and cancer. Such attention is well deserved as TNFα is unique in its engagement of pleiotropic signaling *via* its two receptors: TNFR1 and TNFR2. Extensive research has yielded mechanistic insights into how a single cytokine can provoke a disparate range of cellular responses, from proliferation and survival to apoptosis and necrosis. Understanding the intracellular signaling pathways induced by this single cytokine *via* its two receptors is key to further revelation of its exact functions in the many disease states and immune responses in which it plays a role. In this review, we describe the signaling complexes formed by TNFR1 and TNFR2 that lead to each potential cellular response, namely, canonical and non-canonical NF-κB activation, apoptosis and necrosis. This is followed by a discussion of data from *in vivo* mouse and human studies to examine the differential impacts of TNFR1 versus TNFR2 signaling.

## Introduction

Tumor Necrosis Factor alpha (TNFα) is a central mediator in the immunologic processes of infection control, autoimmunity, allergic disease, as well as the anti-neoplastic activity for which it was named. While too often categorized as simply “pro-inflammatory”, modern research has identified the complex and pleotropic nature of TNFα signaling. However, many gaps in knowledge persist in differentiating the signaling through the two main TNFα receptors (TNFR1 and TNFR2) or how the balance between them may influence downstream consequences. Activation of signaling by TNFα through TNFR1 and TNFR2 initiates a variety of potential outcomes, including cell proliferation, gene activation or cell death. Mediating this variety of cellular responses from just two receptors requires complex control of signal transduction within the cell.

The particular response of a given cell to activation by TNFα is determined by receptor expression and intracellular conditions, such as ubiquitination of the signaling complex and the availability of caspases. Herein, we attempt to summarize the difference in intracellular signaling downstream from TNFR1 versus TNFR2 as well as their specific biologic consequences. In addition, we collate the animal model and human clinical studies which independently assess TNFR activity. Overall, we identify that a greater understanding of this pathway may offer opportunities for novel therapeutic targeting as well as treatment optimization for current TNFα inhibition approaches.

## TNFR Cellular Expression

TNFα receptor expression is central to the cellular response to TNFα and varies by cell type. TNFR1 is a death receptor, as its structure includes a death domain (DD), that is constitutively expressed on most cell types and is activated by TNFα in either its membrane-bound (mTNFα) or soluble (sTNFα) forms (1–3). Following activation by binding TNFα, intracellular signaling *via* TNFR1 is initiated *via* its DD (4).

In contrast, TNFR2 is limited in both its expression and its activation. Expression of TNFR2 is restricted to particular cell types, including, endothelial cells, fibroblasts and subsets of neurons and immune cells (myeloid cells, T- and B-cell subsets) (5, 6). Among the peripheral immune cells, both the percentage of cells expressing, and the number of receptors per cell, is much greater for TNFR2 than TNFR1 (7). TNFR2 is only fully activated by mTNFα, and this receptor lacks the DD that is central to intracellular signaling by TNFR1 (8). The selectivity of ligand, binding sTNFα and/or mTNFα, adds a layer to the regulation of responses that result from activation of TNFR1 or TNFR2. There is a structural basis for preferential binding of mTNFα by TNFR2 based on its rigid proline-rich stalk region, which alters the organization of TNFR2 monomers in the absence of ligand in a manner that inhibits their ability to bind sTNFα (9). Thus, the mechanism of activated pathways and cellular responses differ between the two TNFα receptors.

Further adding to the complexity of TNFR signaling is the production of soluble TNFR1 or TNFR2 during cellular activation and the potential for reverse signaling through binding mTNFα. TNFα or other stimuli (e.g., formyl methionine-leucine-phenylalanine (fMLP), lipopolysaccharide (LPS), GM-CSF) induce proteolytic cleavage of TNFRs by the metalloproteinase TNFα converting enzyme (TACE; also called ADAM17) (10, 11). Soluble receptors can also be generated by alternative splicing of mRNA to produce TNFR2 that lacks the membrane-spanning region (12). These soluble receptors retain their ability to bind TNFα, albeit with lower affinity than their membrane-bound counterparts, thereby reducing the availability of TNFα for receptors that remain membrane-bound (12, 13). When mTNFα is bound by either TNFR1 or TNFR2, reverse signaling can occur. In this case, once TNFR binds mTNFα, it is phosphorylated within the ligand-bearing cell, resulting in NFκB activation (a pathway described in more detail below) (14, 15). Receptor expression and receptor shedding and reverse signaling through membrane-bound ligand provide extracellular regulation for this signaling pathway.

## TNFR1 Activation: Formation of the Core Signaling Complex

**Figure 1:**
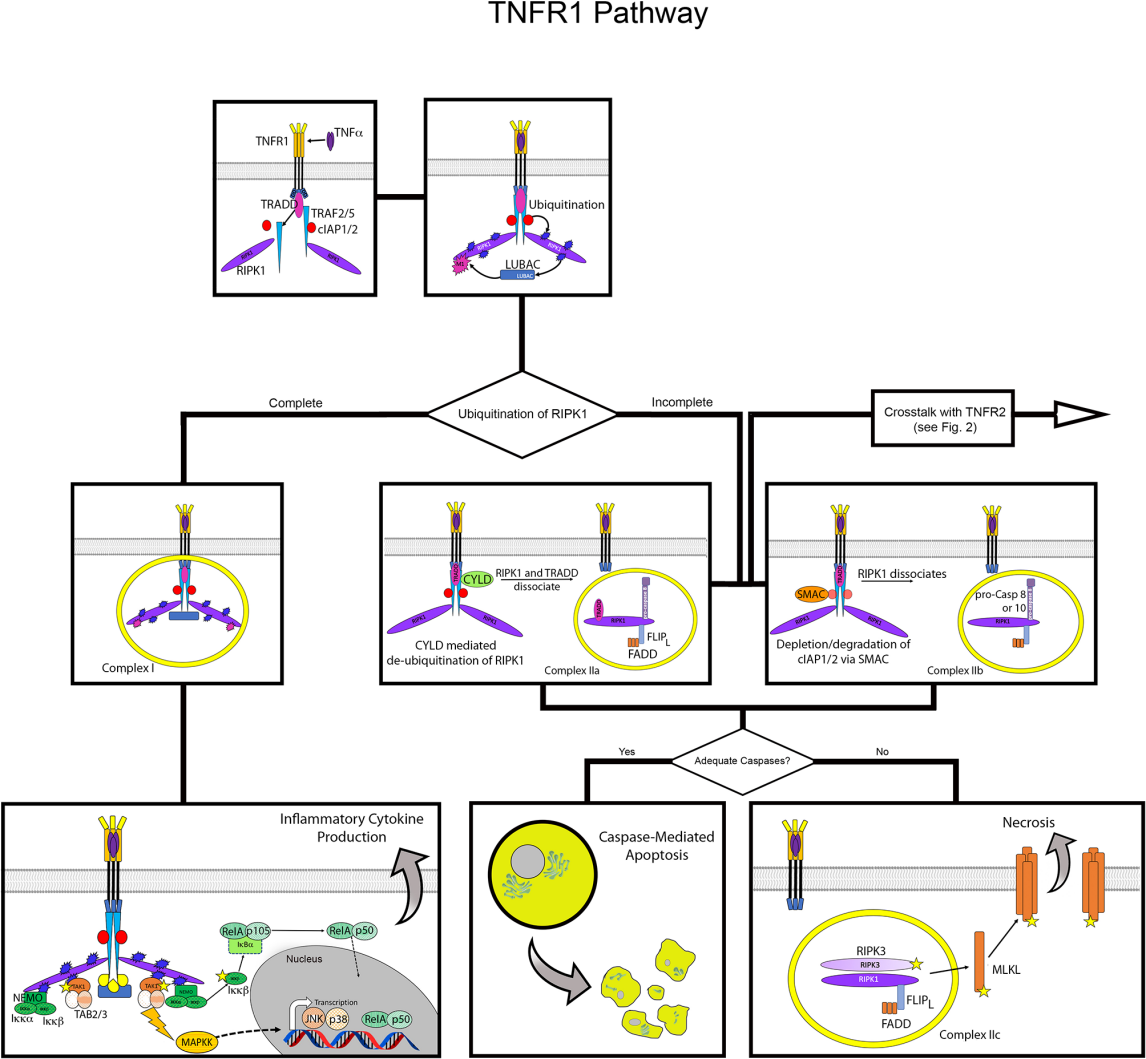
Overview of TNFR1 activation pathway. Flow diagram for TNFR1 activation contrasting the outcomes of inflammatory cytokine production versus cell death. The top panels depict formation of the core signaling complex with recruitment and assembly of TRAF2/5, cIAP 1/2 and RIPK1 at the death domain of TNFR1, and subsequent recruitment of LUBAC. The middle and lower panels show the divergence of potential pathways from formation of the core complex. On the left, complete ubiquitination of RIPK1 results in formation of Complex I, which leads to recruitment of NEMO and TAK1 that activate NFκB and JNK, respectively. On the right, incomplete ubiquitination of RIPK1 leads to formation of complex IIa or IIb, with assembly of FLIP_L_, FADD and pro-caspase 8 or 10, leading apoptosis *via* activation of the latter. Below this, the formation of Complex IIc in the absence of sufficient caspases leads to necroptosis *via* activation of MLKL *via* the necrosome formed by assembly of RIPK1 and RIPK3. Stars indicate phosphorylation, blue bursts represent ubiquitination, purple bursts represent M1 ubiquitination.

When activated by binding TNFα, a core signaling complex is constructed on the cytoplasmic tail of TNFR1. The first step in this process is the trimerization of TNFR1, initiated by contact with TNFα (16). Once TNFR1 forms a trimer, the DD is able to recruit TNFR1-associated death domain (TRADD) (4). TRADD acts as a scaffold in the TNFR1 signaling complex, directing all downstream signaling events as it recruits TNF receptor-associated factor (TRAF) 2, or TRAF5, and receptor-interacting serine/threonine-protein kinase 1 (RIPK1) (17, 18). TRAF2 then provides a platform for recruitment of cellular inhibitor of apoptosis protein (cIAP) 1 and cIAP2 (19, 20) (**Figure 1**; **Supplemental Figure 1**).

The assembly of TRADD, TRAF2 (or TRAF5), RIPK1, and cIAP1/2 forms the core signaling complex of activated TNFR1. From here, the cellular response mediated by TNFR1 is determined by the ubiquitination of RIPK1 and the availability of caspase molecules (21, 22). The TRAF proteins and cIAP1/2 are ubiquitin E3 enzymes that add ubiquitin chains to RIPK1 at multiple locations (20, 23). These ubiquitin chains act as scaffolds for additional factors that lead to the formation of Complex I, which activates NF-κB, JNK, and p38 pathways to induce cytokine signaling and cell survival (20). If the addition of these ubiquitin chains is disrupted, various versions of Complex II can form, leading to apoptotic or necrotic cell death. The details of the formation of these complexes are discussed below.

## Complex I: Activation of NF-κB, JNK, and p38 Pathways Leads to Gene Activation and Survival

The formation of Complex I is stabilized by linear ubiquitination of RIPK1, which allows the recruitment of additional signaling factors (24). Addition of K63, K11, and K48 poly-ubiquitin chains by cIAP1/2 recruits a multimeric complex known as linear ubiquitin chain assembly complex (LUBAC) (25, 26). Complex I is fully activated by attachment of an M1 poly-ubiquitin chain to RIPK1 by LUBAC, completing the formation of the scaffolding network that recruits additional mediators for gene activation (25).

Activation of multiple pathways, specifically NF-κB, JNK, and p38, by Complex I is achieved by parallel assembly of proteins, recruited *via* their ubiquitin-binding domains, to activate TGFβ-activated kinase 1 (TAK1) and inhibitor of IκB kinase (IKK). TAK1 is activated by its recruitment with TAK1 and MAP3K7-binding protein (TAB) 2 and TAB3, with TAB2 and TAB3 binding to poly-ubiquitin chains (27). Alongside the recruitment and activation of TAK1, the IKK complex is recruited by assembly of IKKα, IKKβ and NF-κB essential modulator (NEMO), *via* the ubiquitin-binding domain of the latter (28–30). Once in proximity to each other, TAK1 activates the IKK complex *via* phosphorylation of IKKβ (27, 28).

TAK1 also activates mitogen-activated protein kinase kinases (MAPKKs), which activate JUN NH_2_-terminal kinase (JNK) and p38 pathways (21, 31). The NEMO-dependent activation of the IKK complex results in canonical NF-κB activation, freeing the RelA/p105 heterodimer to undergo proteolytic processing to RelA/p52 and subsequent nuclear translocation (30, 32). Thus, formation of Complex I initiates signaling that induces inflammatory gene activation and cell survival *via* activation of multiple transcription factors. The particular genes induced by TNFα vary widely by cell type, with as many as >5000 genes modulated by the various transcription factors in a highly dynamic manner (33–36). If Complex I does not form upon stimulation of signaling through TNFR1, the pathways for cell survival are not initiated and different versions of Complex II form to mediate cell death *via* apoptosis or necroptosis (37).

## Complexes IIa and IIb: Apoptosis *via* Caspase 8

Returning to the principle that full ubiquitination of RIPK1 is necessary for the formation of Complex I, conversely, the formation of Complex II is dependent on incomplete ubiquitination of RIPK1 (20, 38). When RIPK1 is not fully ubiquitinated, it dissociates from the signaling complex and apoptotic signaling *via* Complex IIa or IIb is initiated (39). There are multiple ubiquitin-modifying enzymes that can act on RIPK1 to facilitate this process.

Formation of Complex IIa is initiated when RIPK1 is de-ubiquitinated by cylindromatosis (CYLD). CYLD provides a negative feedback loop for NF-κB activation, as its expression is induced by NF-κB (40, 41). When CYLD associates with TRAF2, it removes the K63 and M1 poly-ubiquitin chains from RIPK1, thereby allowing RIPK1 to dissociate from the TNFR1 complex (40, 41). Once released into the cytosol, RIPK1 forms Complex IIa by assembling with TRADD, Fas-associated death domain (FADD), FLICE-like inhibitory protein (FLIP_L_), pro-caspase 8 (39). The assembly of these proteins converts pro-caspase 8 to its active form, caspase 8, initiating the apoptosis pathway.

Incomplete ubiquitination of RIPK1 can also be caused by depletion or degradation of cIAP1/2, which results in the absence or reduction of K63 poly-ubiquitin chains added to RIPK1, leading to the formation of Complex IIb (42, 43). Regulation of cIAP1/2 is mediated, in part, by second mitochondria-derived activator caspase (SMAC) (44). The interaction with SMAC causes auto-ubiquitination of cIAPs, leading to their degradation (45). Due to its destabilization in the core signaling complex by the resultant absence of K63-linked ubiquitination, RIPK1 dissociates from TNFR1 as in the start of Complex IIa. It again forms a complex in the cytosol with FADD, FLIP_L_, and pro-caspase 8. Thus, Complex IIb includes the same components as Complex IIa, except it lacks TRADD (21, 31). This assembly also serves to activate pro-caspase 8 to caspase 8, resulting in cell death by apoptosis (39). Activation of caspase 10 has also been linked to Complex IIa and IIb formation (37).

## Complex IIc: Necroptosis in the Absence of Caspase Activity

After activation of caspase 8 in Complex IIa and IIb, RIPK1 and RIPK3 are degraded by the activated caspase in the cytosol (46, 47). However, if there are not sufficient caspases available to perform this inactivation, necroptosis will occur *via* the activity of RIPK1 and RIPK3. The depletion of caspase 8 to induce necroptosis has mainly been established and studied using targeted inhibition of caspase 8 by pharmacological agents such as Z-VAD-FMK (48, 49). This process has also been observed in cells expressing the caspase inhibitor SPI-2, resulting from vaccinia virus infection, which sensitizes cells to TNFα-induced necroptosis in a RIPK1-dependent manner (50, 51). Once caspase 8 is inhibited or depleted, RIPK1 and RIPK3 assemble together in an amyloid-like structure to form the necrosome, and RIPK3 activates mixed-lineage kinase domain-like protein (MLKL) (52–54). Once activated, MLKL oligomerizes and translocates to the plasma membrane where it binds phosphoinositides to cause cell lysis (53).

## TNFR2 Activation: Canonical and Non-Canonical NF-κB Pathways

**Figure 2:**
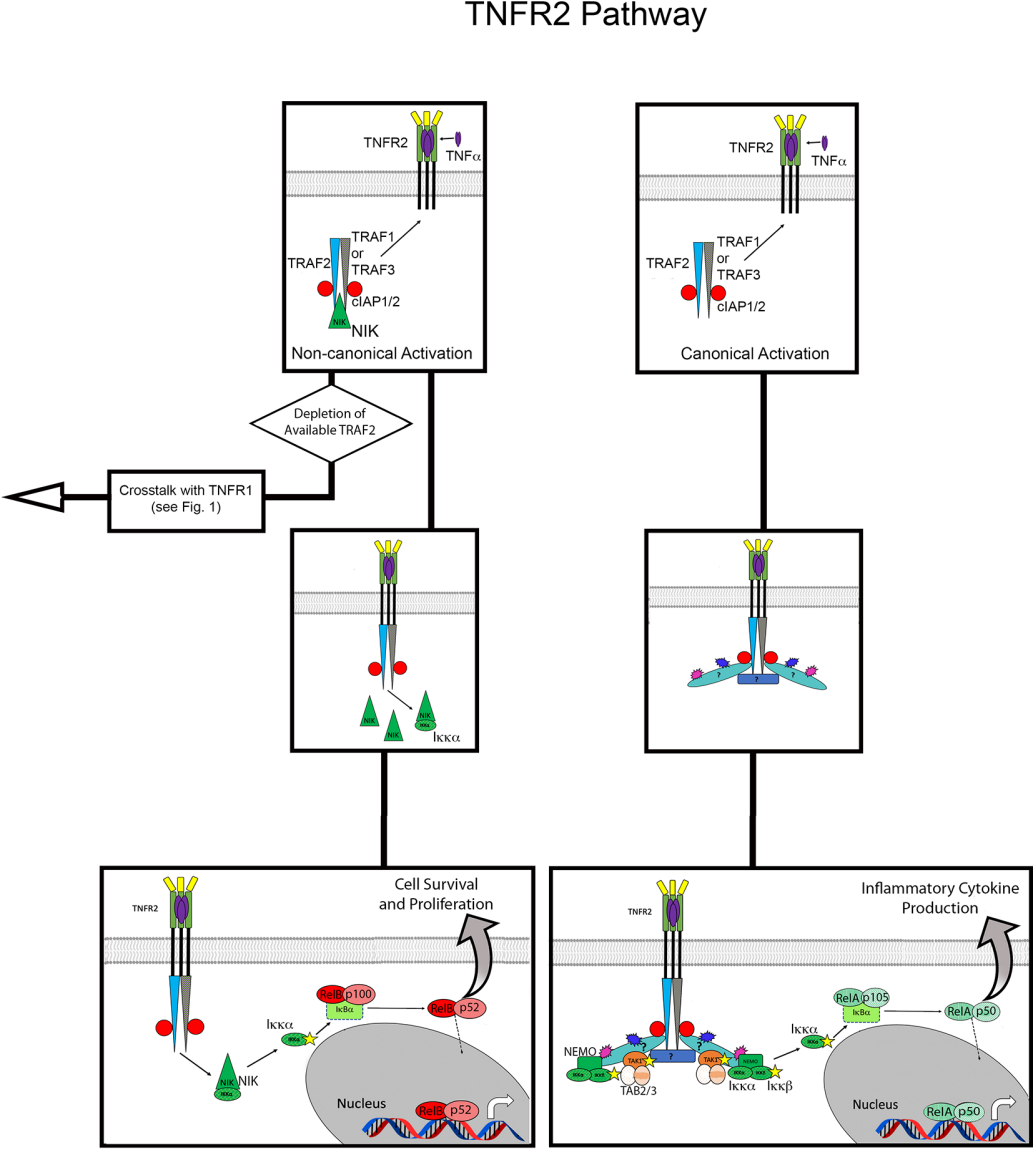
Overview of TNFR2 activation pathway. Flow diagram for TNFR2 activation contrasting the non-canonical versus canonical pathways. The top panels show recruitment of TRAF proteins, cIAP1/2 and NIK to TNFR2 upon binding ligand (mTNFα). On the left, this assembly leads to non-canonical NFκB activation *via* accumulation of NIK. On the right, the pathway of canonical NFκB activation is shown, the details of which are unknown but presumably result from K63 and M1 polyubiquitin chains mediating the recruitment of TAK1 and NEMO. Stars indicate phosphorylation, blue bursts represent ubiquitination, purple burst represent M1 ubiquitination. Question mark in canonical pathways indicate the target of ubiquitination is unknown.

Canonical NF-κB activation is defined by the requirement for NEMO-dependent IKK activation, which frees the RelA/p50 NF-κB heterodimer for nuclear translocation. Thus, NEMO-independent IKK activation is the defining feature of non-canonical NF-κB activation; instead, this pathway involves the accumulation of NF-κB inducing kinase (NIK) to activate IKKα, leading to nuclear translocation of the RelB/p52 heterodimer (32, 33). Although TNFR2 signaling can result in canonical NF-κB activation, this is most often a result of TNFR1 activity (21) (**Figure 2**; **Supplemental Figure 1**).

Activation of TNFR2, which lacks the DD that characterizes signaling by TNFR1, most frequently results in cell proliferation and survival. After binding mTNFα, TNFR2 forms a trimer and directly recruits TRAF2 and TRAF1 or TRAF3, albeit with lower affinity than TNFR1-TRADD (55–57). TRAF2 is central to assembly of the TNFR2 signaling complex, and also recruits cIAP1/2 (19, 20, 58). Although the exact targets remain unknown, it has been demonstrated that the TNFR2 signaling complex includes K63 and M1 poly-ubiquitin chains (59). This suggests a mechanism of canonical NF-κB activation similar to that observed in the TNFR1 signaling complex. Indeed, it has been shown that activation of this pathway by TNFR2 also relies on TAK1 and IKKβ, and the K63 and M1 poly-ubiquitin chains bring these kinases into close proximity with each other (59, 60).

While canonical NF-κB activation tends to be rapid and result in the expression of pro-inflammatory genes, non-canonical NF-κB pathways activate in a sustained manner, on a slower timescale, and promote cell survival and proliferation. This alternative NF-κB pathway utilizes p100, which is produced from the canonical pathway, usually *via* activation of TNFR1 signaling (61). Again, a defining feature of non-canonical NF-κB activation is its induction by NIK (62). In its basal state, NIK is in an inhibitory complex with TRAF 2/3 and cIAP 1/2. When TRAF 2/3 is recruited to TNFR2, the inhibitory complex of NIK is disrupted (63). Activated NIK then accumulates and activates IKKα, allowing p100 to be proteolytically processed to p52, thereby generating the active NF-κB p52/RelB heterodimer (64). Expression of p100 and RelB is potentiated by activation of the canonical NF-κB pathway by TNFR1, which then feeds into the non-canonical activation induced by TNFR2 and results in cell survival and proliferation (61). This signaling synergy between TNFR1 and TNFR2 is an example of crosstalk between the receptors, a type of interaction that is also central to cell death mediated by TNFR2.

## Cell Death *via* TNFR2

As stated in the previous section, TRAF2 is central to the cellular responses generated from the TNFR2 signaling complex. Activation of TNFR2 influences formation of TNFR1 signaling complexes by depleting available TRAF2 for Complex I, as expression levels of TNFR2 can be up to 10-fold higher on cells that express both receptors (65) (**Figure 2**; **Supplemental Figure 1**). Additionally, TRAF2 can be degraded by its interaction with cIAP1/2. This depletion and degradation of TRAF2 has two major consequences: promoting the non-canonical NF-κB pathway, described above, and inhibiting canonical NF-κB activation *via* TNFR1 Complex I (61, 64). The reduction of TRAF2 inhibits recruitment of cIAP1/2 to the TNFR1 signaling complex, thereby allowing formation of Complex IIa, IIb or IIc. Thus, cell death mediated by TNFR2 is a result of crosstalk, where activity of TNFR2 indirectly influences the signaling complexes that can form at TNFR1. In TNFR signaling, cell survival, proliferation and death are a matter of quantitative balance between TNFR1 and TNFR2 and the key components of their respective signaling complexes.

## TNFR Influence on Epithelial and Endothelial Cells

Epithelial-to-mesenchymal transition (EMT) is a pathway heavily mediated by TNFR in endo- and epithelial cells. EMT is an essential process for normal tissue repair; epithelial cells take on a migratory mesenchymal phenotype *via* a complex modulation of adhesion proteins and intracellular filaments (66, 67). During wound healing, polarization of cells on the leading edge of the damaged tissue activates EMT-related pathways to fill the cellular gaps through a combination of migration and proliferation (67, 68). Subsequent mesenchymal-to-epithelial transition (MET) re-epithelializes the damaged area (67, 68). While both receptors are involved in the inflammatory and anti-infection responses (69), TNFR2 is central for EMT and cell proliferation (70). The potential for differing impacts of TNFR activation in MET is currently unknown. TNFα-related EMT can be further modified by phospholipid exposure (71, 72) as well as co-stimulation of Toll-Like Receptors (TLR) and neurotransmitters like nicotine and serotonin (73–78).

Furthermore, the consistent, direct contact between neighboring epithelial cells heightens the potential influence of mTNFα. While genetic deletion of TACE/ADAM17 does not appear to impact bacterial responses in peripheral immune cells (79), TACE/ADAM17 knockout mice develop a microbiome-dependent, eczematous phenotype (80, 81). Endothelial-to-mesenchymal transition (EndoMT) is also a process of developing a migratory phenotype important in vascularization. Although EndoMT is known to be influenced by TNFα (82, 83), differential impacts of the receptors of membrane bound status of TNFα remain unelucidated.

## Differential TNFR Activity in Mouse Models

**Table 1** details all identified mouse models in which TNFR1 and TNFR2 were assessed independently. In brief, neurologic models have demonstrated even more complex effects of selective TNFR deletion; in EAE, TNFR2 is protective on microglia but deleterious on monocytes and macrophages whereas TNFR1 appears harmful in all contexts due to reducing the blood brain barrier function against cell infiltration into the CNS (84, 105, 117). In mouse models of pain, deletion of TNFR1 lead to reduction of mechanical pain as well as NMDA activation of lamina II neurons (86, 87, 134). Early-phase hyperalgesia responses to heat after exposure to complete Freud’s adjuvant (CFA) showed dual dependence on TNFR1 and TNFR2, whereas late phase reactions were TNFR1 exclusive (86). CFA injection into mouse footpads led to an increase in TNFR2 mRNA in the spinal cord (86).

**Table 1:** Summary of mouse model and clinical association data for TNFR1 versus TNFR2.

Mouse Models		
	TNFR1^−/−^ (or blockade)	TNFR2^−/−^ (or blockade)
**Outcomes Improved**	**Neuro** Experimental autoimmune encephalitis (EAE; model of acute demyelination disease) *via* reduced immune cell infiltration across blood brain barrier (84) Retinal detachment induced photoreceptor degeneration (85)^$^ Early phase heat hyperalgesia after CFA injection (86)^$^ Late phase heat hyperalgesia after CFA injection (86)* Mechanical pain (87) Pain mediated by NMDA activation of lamina II neurons (86) **Gastrointestinal** High fat induced liver steatosis (88) Total peripheral nutrition (TPN) induced epithelial barrier function loss (89) **Infection/Inflammation/Allergic** *Staphylococcus aureus* sepsis *via* T-cell anergy (90) Sterile endotoxemia (91) LPS induced bone loss (92) LPS-induced systemic apoptosis of non-granulocyte bone marrow cells (93) Cecal ligation and puncture model of polymicrobial sepsis (94)* Loss of small in olfactory dysfunction and chronic rhinitis models (95) *Chlamydia pneumoniae* induced atherosclerosis (96)^$^ Allergic contact dermatitis model (reduced allergen uptake but not migration of dendritic cells (DC) into lymph nodes) (97)^$^ **Cardio-renal** Transverse aortic constriction model of cardiac stress *via* Stat3 (98) Mesenchymal stem cell as treatment of cardiac ischemia (99)* Femoral artery ligation model of ischemia (100)* Heart failure model (101)* Thrombosis model (102)* **Endocrine** Adrenalectomy model of Addison’s (103) **Pulmonary** Transgenic model of spontaneous COPD; TNR1^−/−^ improved more than TNFR2^−/−^ mice (104)	**Neuro** EAE if selectively deleted in monocytes/macrophages (105) Retinal detachment induced photoreceptor degeneration (85)^$^ Neuronal loss (but not motor function) in SOD1-G93A model of ALS (106) Early phase heat hyperalgesia after CFA injection (86)^$^ **Gastrointestinal** Rhesus rotavirus-induced biliary atresia (107) Trinitrobenzene sulfonic acid colitis (108)* DSS colitis (109)* **Infection/Inflammation/Allergic** Allergic contact dermatitis model (reduced DC migration but not allergen uptake) (97)^$^ Chronic TNFa induced inhibition of TCR-dependent, but not TCR independent T-cell activation (110) *Chlamydia pneumoniae* induced atherosclerosis (96)^$^ **Endocrine** Adrenalectomy model of Addison’s (103) **Pulmonary** Transgenic model of spontaneous COPD; TNR1^−/−^ improved more than TNFR2^−/−^ mice (104) **Cancer** Breast cancer cell line challenge (111)
**Outcomes Worsened**	**Neuro** Post-exercise recognition memory (112) HSV-1 ocular infection (113) **Gastrointestinal** Trinitrobenzene sulfonic acid colitis (108)* DSS colitis (109)* **Infection/Inflammation/Allergic** *Roseomonas mucosa* treatment of atopic dermatitis (114)^$^ **Cardio-renal** Angiotensin II infusion induced hypertension; TNFR2 not evaluated (115)	**Neuro** Theiler murine encephalomyelitis virus epilepsy model (116) EAE if selectively deleted in microglia (105) or whole (117) **Gastrointestinal** Cell mediated colitis (118) **Infection/Inflammation/Allergic** Cecal ligation and puncture model of polymicrobial sepsis (94)* *Roseomonas mucosa* treatment of atopic dermatitis (114)$ **Cardio-renal** Mesenchymal stem cell as treatment of cardiac ischemia (99)* Femoral artery ligation model of ischemia (100)* Heart failure model (101)* Thrombosis model (102)*
**Human Data**		
	**TNFR1-specific**	**TNFR2-specific**
**Associations**	**Neuro** Elevations a/w worse sleep in patients with depression; reductions a/w improved sleep and symptoms after infliximab treatment (119) **Gastrointestinal** Elevations predict post-liver transplant need for dialysis and overall mortality (120) **Infection/Inflammation/Allergic** Increased expression on T-cells of patients with atopic dermatitis (121) Reduction a/w clinical improvement of lupus after atorvastatin therapy (122) Higher expression on alveolar macrophages in hypersensitivity pneumonitis (123)* Nebulized anti-TNFR1 reduced inflammation in pulmonary endotoxin challenge (124) **Endocrine** In diabetics presenting to an ER with shortness of breath, elevations associated with short-term mortality, HR, BMI, and renal function; was not significant when adjusting for CRP (125) Elevations predictive of renal disease in diabetics (126–128), **Cancer** Elevations a/w worse outcomes in patients with GVHD after a BMT with ablative conditioning (129) **Monogenic Disorders** [all reviewed in (130)] TNF receptor-associated periodic syndrome (TRAPS); mutations in *TNFRSF1A* Haploinsufficiency of A20 (HA20); mutations in *TNFAIP3* OTULIN-related autoinflammatory syndrome (ORAS); mutations in *FAM105B* LUBAC deficiency; mutations in *HOIP/HOIL* or *SHARPIN* RIPK1 associated immunodeficiency and autoinflammation; mutations in *RIPK1* X-linked ectodermal dysplasia and immunodeficiency (X-EDA-ID); mutations in *IKBKG/NEMO* RELA haploinsufficiency; mutations in *RELA*	**Infection/Inflammation/Allergic** Higher expression on lymphocytes in hypersensitivity pneumonitis (123)* **Cancer** Tissue expression higher in breast cancer cells versus healthy breast tissue (131) **Monogenic Disorders** ADAM17/TACE deficiency a/w reduced LPS-stimulated TNFa and sTNFR2 in PBMC (132, 133),

Mouse models included in the table indicate selective deletion or antibody blockade of TNFR1 or TNFR2. Models using only TNFR double knockout mice were not included. Human data for diseases associated with changes in one TNFR (serum or tissue) are shown. *indicates opposing effects of TNFR deletion (for example, deletion of TNFR1 worsening outcomes while deletion of TNFR2 improving them); ^$^indicates similar effects of TNFR deletion where both were assessed independently. LPS, lipopolysaccharide; DSS, dextran sodium sulfate; COPD, chronic obstructive pulmonary disorder; HSV-1, herpes simplex virus 1; TCR, T-cell receptor; ER, emergency room; HR: heart rate; BMI, body mass index; CRP, C-reactive protein; GVHD, graft versus host disease; BMT, bone marrow transplant; CFA, complete Freud’s adjuvant; LPS, lipopolysaccharide; PBMC, peripheral blood mononuclear cells; a/w, associated with.

Gastrointestinal models identified TNFRdko and TNFR1^−/−^ mice have worse outcomes during DSS colitis (135), however selective deletion of TNFR2 improves outcomes (109). While TNFR2 deletion improved DSS colitis (109), TNFR2 is protective in cell-mediated colitis models, potentially through its role in fostering the expansion and stability of regulatory T cells (Tregs) (118) and vitamin D dependent tolerogenic dendritic cells (136, 137).

Opposite, but similarly discordant effects were seen in thrombosis models where TNFR2^−/−^ mice display worse outcomes and TNFR1^−/−^ mice were protected (102). Additional cardio-renal models including heart failure and ischemia also displayed opposing influence of TNFR1 versus TNFR2 (99–101). Models of adrenal insufficiency (103), chronic obstructive pulmonary disease (COPD) (104), and breast cancer (111) each identified a pathogenic role for TNFR2.

The lone infection model differentiating TNFR impacts uncovered in our search demonstrated TNFR2-mediated protection and TNFR1-mediated harm during polymicrobial sepsis resulting from cecal ligation and perforation (94). However, the importance of TNFR2 in wound repair may confound any infection-control conclusions of this surgical model. Meanwhile, LPS mediated effects on infection and inflammation appear to operate through TNFR1 (91–93), while off target effects of inflammation (such as allergic sensitization and *Chlamydia* induced atherosclerosis) were mediated by TNFR2 (96, 97). Both allergic rhinitis-associated and neurogenic olfactory dysfunction are mediated by TNFR1 (95). Our recent publication demonstrated that the modeled treatment efficacy of topical, commensal *Roseomonas mucosa* for atopic dermatitis operates through TNFR2, however both receptors are essential for modeled therapeutic benefit (114). Consistent with prior literature, the TNFR2-mediated effects also demonstrated a role for neurologic and innate signaling; TNFR2 potentiation by optimal TLR5 and nAChR activation were essential to modeled treatment outcomes (114). Therefore, while *in vivo* evidence is limited, the therapeutic potential of selective TNFR blockade remains an under-elucidated topic.

## Viral TNF Blockade

Several examples of virus encoded TNFα-binding proteins have been described in the literature (138). T2 have been identified in the leporipovirus myxoma virus (M-T2) and the Shope fibroma virus (S-T2) (139, 140). While extracellular M-T2 inhibition is species-limited to rabbits, S-T2 can also inactivate human TNFα. However, in the intracellular form, M-T2 can inhibit TNFR-1–mediated cell death by directly binding to human TNFR1 (141). The poxvirus produces several TNFR decoy receptors: CrmB and CrmD bind TNFα as well as leukotriene alpha (142, 143); CrmC and CrmE exclusively bind TNFα (142, 144, 145). Additionally, the YLDV and tanapox viruses produce the decoy receptor 2L, an major histocompatibility complex I (MHC-I) analogous protein which also competitively binds TNFa (146). Other examples of direct viral influence over the TNFα pathway include the SARS-CoV1-S protein, which activates TACE to cause increased shedding of ACE2 and increased solubilization of TNFα (147). These viral TNFα modulation strategies present intriguing opportunities for therapeutic development.

## Pharmacologic TNF Blockade

The predominant TNF blockade strategy is through antibody-mediated inhibition: chimeric (infliximab); fully humanized (adalimumab and golimumab); and variable binding regions fused to a Peg moiety (certolizumab-pegol). Alternatively, etanercept is a dimer of recombinant TNFR binding regions fused to the constant region of a human IgG1 antibody (148). Although these medications have been revolutionary in the treatment of autoimmune and autoinflammatory diseases, their results do not inform TNFR actions given that they bind the TNF ligand and thus preclude signaling through both receptors (148).

Pentoxifylline is an oral medication which inhibits the release of TNFα. While the mechanism remains to be elucidated, the proposed mechanism is *via* influence over phosphodiesterase (PDE) and cyclic (cAMP) (149), further implicating lipid metabolism in the control of TNFα (71, 72). Of note, current TNFα inhibition strategies are unlikely to block the impacts of mTNFα. Given the greater binding affinity of the current anti-TNF agents for sTNFα (150, 151), tissue-level analysis may be needed to determine if meaningful neutralization of mTNFα can be achieved by any current anti-TNFα therapy.

Although TNF blockade is now a routine part of clinical medicine, details of differential impacts of TNFR signaling in humans have larger gaps in knowledge than murine models. As stated, all current anti-TNF treatments are non-selective, and thus, only a paucity of clinical evidence for selective blockade exists (**Table 1**). While several anti-TNFR1 antibodies have been used *in vitro* (137), only one anti-TNFR1 antibody has been studied in humans; a nebulized formulation reduced inflammation and endothelial injury in healthy subjects that underwent an inhalational LPS challenge (124). The drugs thalidomide and cyclophosphamide have reportedly TNFR2-specific inhibitory effects, but confirmatory evidence is thus far limited to the effects on Treg differentiation; and, the side effect profile for thalidomide does not favor clinical use outside of cancer treatment (137).

## Soluble and Tissue TNFR Expression in Inflammation

While interventional evidence is lacking, several publications demonstrate disease associations with differential TNFR effects, particularly for serum levels of sTNFR (119–123, 125–129, 131). Intriguingly, the concentrations of both sTNFR1 and sTNFR2 follow circadian rhythms that run about 1 h ahead of the well-established cortisol fluctuations (152). Thus, time of day may be an important confounder for clinical studies that aim to compare sTNFR levels in disease. However, a far more concerning confounder is the possibility of acute phase reactions. Several examples of treatment responsive, equivalent elevations in both sTNFR1 and sTFNR2 have been described in tuberculosis (153), multiple sclerosis (154), schizophrenia (155), Alzheimer’s disease (156), hyperlipidemia (157), severe burns (158), and after dietary challenge with transfat (159). A study evaluating predictors of infarction in heart disease may provide the most illustrative example for the possibility of sTNFR1 and sTNFR2 being general inflammatory markers; elevations of both sTNFR1 and sTNFR2 were highly predictive of future cardiac events but lost statistical significance when adjusted for levels of the more commonly deployed inflammatory marker CRP (160). Similarly, tissue levels of both TNFR1 and TNFR2 are elevated in psoriasis and decrease with successful anti-TNFα treatment (161). However, not all patients respond to anti-TNFα treatment and the risk of infectious complications must be considered whenever TNF-inhibitors are prescribed (162).

## Differential Soluble and Tissue TNFR Associations in Disease

While sTNFR1 and sTNFR2 may simultaneously track with general inflammation, a unique differential association is found in obesity, where elevations are seen in serum TNFα but not sTNFR1 or sTNFR2 (163). More distinguishing associations between disease and TNFR1 have been reported compared to TNFR2, however several studies failed to assess sTNFR2 levels (164, 165). Overall, the most robust clinical association for sTNFR1 is for predicting future renal disease in diabetics (126–128). Elevated sTNFR1 (but not sTNFR2) was associated with worse outcomes when presenting to an emergency room with shortness of breath, but also lost statistical significance when adjusting for CRP (125). Associations unique to sTNFR1 were also seen with treatment-responsive lupus (122), sleep disruption in depression (119), worse outcomes in GVHD after ablative-conditioned BMT (129), and mortality and dialysis needs for post-liver transplant patients (120). Two studies have assessed the expression of TNFR1 versus TNFR2 on immune cells (121, 123), compared to healthy controls: patients with atopic dermatitis have increased expression of TFNR1 on their T-cells (121); patients with hypersensitivity pneumonitis have elevated TNFR1 expression on alveolar macrophages but elevated TNFR2 on peripheral lymphocytes (123). Furthermore, human cell culture models challenged with *R. mucosa* confirmed the importance of the TNFR2 related pathways identified in our mouse models (114).

## Monogenic Disorders of TNFR Signaling

Although many gaps in knowledge for common diseases, monogenic disorders related to TNFR signaling can provide valuable insights. The most demonstrative of these disorders in TNF receptor-associated periodic syndrome (TRAPS) resulting from autosomal dominant mutations in *TNFSF1A*. Although the exact mechanism is unclear, aberrant TNFR1 signaling, intracellular accumulation of misfolded proteins, and constitutive immune activation have been implicated in the pathogenesis (166). Patients most commonly experience severe abdominal pain, arthralgias, and myalgias (167). Although the anti-IL-1 agent anakinra has become the preferred treatment in TRAPS, etanercept has been used (130). Multiple other monogenic autoinflammatory syndromes are associated with the intracellular complexes downstream of TNFR1, including haploinsufficiency of A20 (HA20), LUBAC deficiency, RIPK1 deficiency, OTULIN-related autoinflammatory syndrome (ORAS), RELA haploinsufficiency, and X-linked ectodermal dysplasia and immunodeficiency (X-EDA-ID); each of these has been expertly detailed in a recent review (130).

In 2011, a syndrome including inflammatory skin disease and recurrent infections was linked to autosomal recessive mutations in ADAM17/TACE in two siblings. One sibling died at age 12 of parvovirus B19-associated myocarditis. However, while the other was shown to have exaggerated production of IL-1β and IL-6 in response to lipopolysaccharide (LPS) challenge, he was described as living a “relatively normal life” despite continued recurrent infection (132). Subsequently, another patient with ADAM17/TACE mutations resulting in inflammatory skin disease, recurrent infections, and fatal sepsis was identified (133). Studies into LPS responses in the patient’s peripheral blood mononuclear cells (PBMC) revealed reduced production of TNFα and sTNFR2 (133).

### Discussion

Data from cell cultures, animal models, and humans strongly suggests the balance between TNFR1 and TNFR2 activity has meaningful impacts on health and disease. Unfortunately, mouse models which independently assess TNFR influence are uncommon in the literature and often lack confirmatory validation between research groups. Similarly, clinical studies that independently assess sTNFR levels are typically small and lack tissue-level verification. Clinical utility of anti-TNF treatments should not preclude investigation into differential impacts of TNFRs, given that more targeted blockade could lead to further optimization of clinical benefit, or a reduction in side effects.

The current literature on the importance of the TNFRs suggests actionable needs for future research to assess sTNFR1 and sTNFR2 during studies of diseases in which TNFα is known to play a role. Ideally, this research could be bolstered by tissue staining for receptor expression, mTNFα concentration, and/or transcription factor post-translational modifications. Furthermore, functional assessments such as the scratch assay should be considered wherever a role for TNFα-modulation of epithelial or endothelial cellular activity is suspected.

However, a notable portion of the differences in downstream signaling through TNFR1 versus TNFR2 is neither transcriptionally regulated nor governed directly by receptor expression. Thus, research into TNFα-modulated diseases using transcript- or proteomics approaches may require direct assessment of the ubiquitination and phosphorylation of signal transducers of the TNFR pathway. Similarly, investigations into disease states or experimental conditions that reveal involvement of pathways that modulate TNFR activity, such as lipid metabolism or neurotransmitter signaling, may be enhanced by directed inquiry into possible TNFR-dependent impacts.

## Author Contributions

PG and IM contributed equally to writing the manuscript and editing the text. IM made the figures and table, with the assistance of PG. All authors contributed to the article and approved the submitted version.

## Funding

This research was supported by the Intramural Research Program of the NIH and NIAID.

## Conflict of Interest

The authors declare that the research was conducted in the absence of any commercial or financial relationships that could be construed as a potential conflict of interest.
