# Supplementary figures and images for: Tumor Necrosis Factor Receptors: Pleiotropic Signaling Complexes and Their Differential Effects
Source: Front Immunol. 2020 Nov 25;11:585880. doi: 10.3389/fimmu.2020.585880 (PMC7723893; doi:10.3389/fimmu.2020.585880)

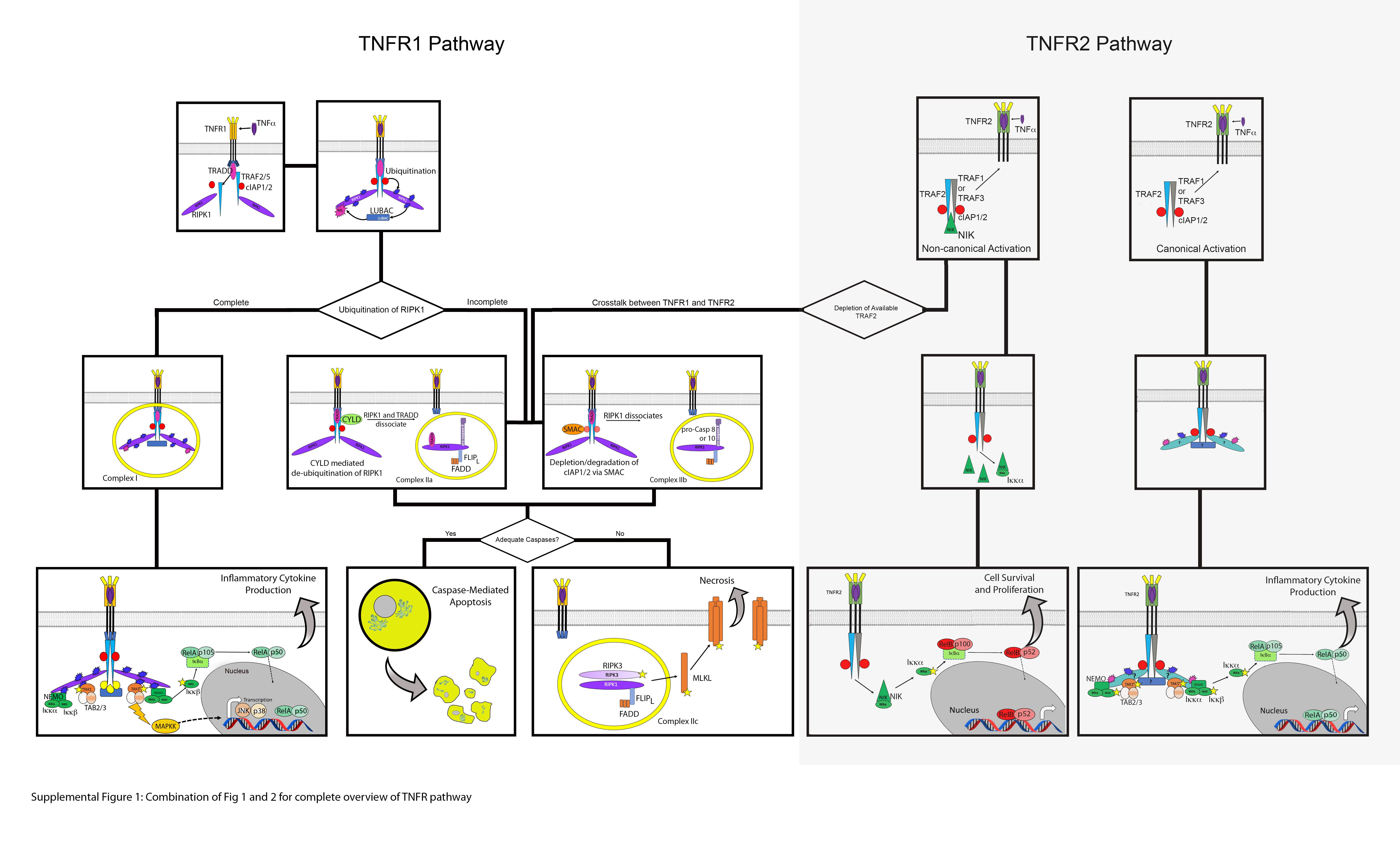

Supplement: Supplementary file 1 [file Image_1.jpeg]
